# Supplementary material for: Acceptability of extending HPV-based cervical screening intervals from 3 to 5 years: an interview study with women in England
Source: BMJ Open. 2022 May 4;12(5):e058635. doi: 10.1136/bmjopen-2021-058635 (PMC9073390; doi:10.1136/bmjopen-2021-058635)
Supplement: Supplementary data [file bmjopen-2021-058635supp002.pdf]

Women's opinions on the length of time between cervical screenings (interview study)

TOPIC GUIDE

[START RECORDING]

1. INTRODUCTION (5 mins)

- Intro to researcher
- Acknowledgement of the current pandemic situation
- Study topic and funder, aims/objectives of study
- Explanation of confidentiality/anonymity
- Explanation of recording, duration of interview, nature of discussion, outputs/audiences for findings, data security and rights.
- Reminder of £50 payment, Saros will pay this within a week
- Go through consent process (reading out points from information sheet – should have been shared in advance so they had time to read and consider it)
- Ask whether they have any questions
- Ask whether they are happy to continue and collect consent

[START NEW RECORDING]

2. UNDERSTANDING OF AND FEELINGS REGARDING CERVICAL SCREENING (5 mins)

- Understanding of process/purpose of cervical screening
- Broad feelings/sentiments regarding cervical screening programme and smear tests

3. ACCEPTABILITY OF CURRENT SCREENING INTERVAL (10 mins)

- Awareness of current screening interval

Script: At the moment, the screening interval is 3 years for women in your age-group: from 25-49 years.

- Views re: <u>current 3-year frequency</u> in terms of:
  - How it makes them feel (affective attitude)
  - Amount of effort or how easy/difficult it is to be screened 3-yearly (burden)
  - How effective it is (perceived effectiveness)
    Prompt: could it be shorter or longer?
  - Fit with personal values (ethicality)
  - Does it make sense; why they think the interval is 3 years (intervention coherence)
  - Any disadvantages to being screened 3-yearly (opportunity costs)
  - Confidence in ability to get screened every 3 years (self-efficacy)

3. ACCEPTABILITY OF NEW SCREENING INTERVAL (15 mins)

Script: We've talked about the 3 year screening interval for women aged 25-49 years. From next year, the screening programme is going to change so that women with normal screening results will be invited for their next screen in 5 years instead of 3.

- Initial reactions
  - Thoughts and feelings
  - Questions and concerns – what would they want to know about the change

Script: I am now going to give you a bit more background to help explain why the interval is changing:

There are three main steps in the development of cervical cancer. The first is infection with HPV, which is a virus. If the immune system doesn't clear the virus, it can cause the second step, abnormal cells. And thirdly, if the abnormal cells aren't treated, they can turn into cancer. Cervical screening

Women's opinions on the length of time between cervical screenings (interview study)

used to look for abnormal cells (the second step), but in 2019, cervical screening changed so samples are now tested for the HPV virus. Finding HPV picks up the first step in that process, at a really early stage. This means it is now considered safe to screen all women every 5 years.

[Check in] How this information influences their initial thoughts

IF NEEDED: Most people will get the virus at some point in their life. It is spread through close skin to skin contact during any type of sexual activity with a man or woman. HPV can stay in the body for many years. It can stay at very low or undetectable levels and not cause any problems.

- Now that you have listened to this information…
  Views re: new 5-year frequency in terms of:
  - How it makes them feel (affective attitude) Does information change their initial reactions
  - Amount of effort or how easy/difficult it is to be screened 5-yearly (burden)
  - How effective it is (perceived effectiveness)
    Prompt: will 5-yearly screening find the women who need further tests and checks?
  - Fit with personal values (ethicality)
  - Does it make sense (intervention coherence)
  - What do they have to give up to be able to get screened 5-yearly (opportunity costs)
    Prompt: What would you feel you were missing out on if you had 5-yearly screening instead of 3-yearly?
  - Confidence in ability to get screened every 5 years (self-efficacy)
  - What else do you need to know?

4. RESPONSES TO KEY MESSAGES (15 mins)
Script: I am now going to read six messages that we think help to further explain the change in screening intervals. After each one I am going to ask what you think and whether you find the message helpful or not. You may want to rephrase the message in a way that makes sense to you, or say if something is confusing.

OK, ready (check), so the first message is…

1. If a woman has a negative HPV result, it's very rare for her to develop abnormal cells in less than 5 years [Prompt response]

2. If a test does not indicate (show) you have a HPV infection, it is safe to wait five years between tests. [Prompt response]

3. An HPV test is more accurate than the old smear test. [Prompt response]

4. Women aged 50-64 years already have cervical screening at 5-yearly intervals [Prompt response]

5. Screening more often than needed can lead to unnecessary treatment and worry [Prompt response]

6. Having screening 3 years after a negative HPV result is too soon for any problems to have developed. [Prompt response]

Thanks you, can you think of any other messages you would want to have?

5. CONCLUSION (5 mins)
- Thank participant for their time.
- Reiterate confidentiality, anonymity, right to withdraw from participation.
- Any further questions – contact research team by email. Signpost to support.
